# A Flexible Tactile Sensor with Irregular Planar Shape Based on Uniform Electric Field

**DOI:** 10.3390/s18124445

**Published:** 2018-12-15

**Authors:** Youzhi Zhang, Jinhua Ye, Haomiao Wang, Shuheng Huang, Haibin Wu

**Affiliations:** School of Mechanical Engineering and Automation, Fuzhou University, Fuzhou 350116, China; zhangyouzhi606@gmail.com (Y.Z.); yejinhua@fzu.edu.cn (J.Y.); wanghaomiao.cn@gmail.com (H.W.); shuhenghuang.cn@gmail.com (S.H.)

**Keywords:** tactile sensor, irregular shape, uniform electric field, flexible

## Abstract

Tactility is an essential perception for intelligent equipment to acquire external information. It can improve safety and performance during human-machine interactions. Based on the uniqueness theorem of the electrostatic field, a novel flexible film tactile sensor that can detect contact position and be made into any plane shape is proposed in this paper. The tactile sensor included an indium tin oxide (ITO) film, which was uniformly coated on the polyethylene terephthalate (PET) substrate. A specially designed strong conductive line was arranged along the edge of the flexible ITO film, which has weak conductivity. A bias excitation was applied to both ends of the strong conductive line. Through the control of the shape of the strong conductive line, a uniform electric field can be constructed in the whole weak conductive plane. According to the linear relationship between position and potential in the uniform electric field, the coordinate of the contact position can be determined by obtaining the potential of the contact point in the weak conducting plane. The sensor uses a three-layer structure, including an upper conductive layer, an intermediate isolation layer, and a lower conductive layer. A tactile sensor sample was fabricated. The experiment results showed that the principle of the tactile sensor used for the contact position detection is feasible and has certain precision of position detection. The sensor has good flexibility, and can be made into any plane shape, and has only four wires. It is capable of covering large areas of robot arms, and provides safety solutions for most robots.

## 1. Introduction

With the rapid development of robot technology, intelligent robots are gradually entering the human world [1,2]. Sensors are a prerequisite for intelligent robots and the key to the safe interaction between robot and human or environment. Tactile sensors, which are suitable as a robot “electronic skin,” can sense the change in environment and obtain real-time touch information so that the robot can avoid collisions or purposefully interact with humans [3,4].

Tactile sensors, as the “electronic skin” of robots, usually require flexibility to be able to cover large areas of the robot surface, and can detect the contact force. The sensor should be easy to manufacture, low cost, with good robustness, and long service life [5]. These design requirements pose serious challenges to the development of tactile sensors for robotic “electronic skin.”

Normally, tactile sensors can be divided into the array structure and the non-array structure. With the development of new materials and information technology, array tactile sensors have gradually formed many types, such as piezoresistivity [6,7], capacitance [8,9], piezoelectric [10,11], Magnetoresistance [12], optical [13], etc. For instance, Shin et al. [14] developed an active-matrix pressure-sensitive graphene FET array with air-dielectrics for sensing tactile pressure. The sensor had a wide pressure sensing range from 250 Pa to approximately 3 MPa, high pixel densities, and fast response time. Mohammad et al. [15,16] designed a micro-tactile sensor array that could be integrated within MIS graspers. The sensor was capable of measuring grasping forces, locating the force position as well as estimating the contact object softness by using piezoelectric polyvinylidene fluoride (PVDP) as the sensing element. Overall, there have been many studies on the tactile sensor array structure, and many results have been achieved. However, the tactile sensor array has some drawbacks, such as complex structures and signal processing circuits, discontinued signal detection, poor real-time signal processing, which has limited its large-scale application in robots.

The non-array tactile sensor uses a single sensing unit to monitor the pressure, position, surface texture, temperature or humidity for the whole surveillance area. The non-array sensor should be flexibly attached to any irregular surface of the object. This makes the development of a non-array tactile sensor quite difficult. For instance, Dana et al. [17] proposed an artificial skin that detected the position and speed of a slipping object using a single force sensor. The sensor had an average speed sensing resolution of 10 mm/s, an average position sensing resolution of 15 mm, and was robust to various grip conditions. Pan et al. [18] proposed a flexible tactile sensor fabricated with conductive fabric that could detect contact position. The sensor was a multi-layered structure and could be used to detect the contact position in a large area. There were fewer leading wires and the signal processing is simple. However, the position detection of the sensor was still discrete. Wu et al. [19] introduced a tactile sensor based on a planar electric field. The sensor was flexible and had only four wires, which could be used for the position detection of a large area, but the shape of this kind of sensor could only be rectangular. Vincent et al. [20] presented a tactile sensor made from conductive ink and piezoresistive conductive rubber. The sensor could realize multi-point contact position detection, but the resolution was low. The tactile sensor, as the “electronic skin” of the robot, usually needs to cover the whole body of the robot. Since the outer surface of the robot is irregular, it is necessary to develop tactile sensors with customized shapes corresponding to the robot body surface. Wu et al. [21] designed a flexible annular sectorial sensor with a five-layer structure based on constant electric field. The sensor could be wrapped on the surface of a truncated cone-shaped link for detecting contact position. However, due to its working principle, the sensor could only be fabricated into sectorial shapes, but not into arbitrary irregular shapes. Visentin et al. [22] proposed a tactile sensor fabricated with conductive fabric based on electrical impedance tomography (EIT). The sensor could be made into different shapes and used for multi-point contact position detection. Zhang et al. [23] painted conductive paint on the surface of three-dimensional objects. They applied electrical impedance tomography (EIT) to detect the surface contact position of 3D objects. However, it had a complex algorithm, the hardware system requirements were also high, and the spatial resolution was limited.

In this paper, a new non-array tactile sensor that can be used for continuous contact position detection and made into any plane shape was proposed. The sensor had a three-layer film structure and only four wires. The sensor was flexible and could be used for full-body robot clothing.

## 2. Principle of the Tactile Sensor

### 2.1. Structural Model of the Tactile Sensor

**Figure 1:**
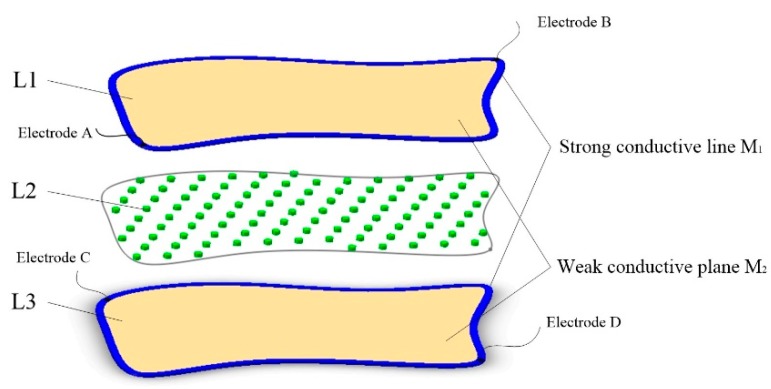
Structure of the tactile sensor.

The proposed tactile sensor consists of three layers, labeled L1–L3, respectively, as shown in Figure 1. The upper layer L1 and lower layer L3 are both the conductive planes composed of the strong conductive line M_1_ and weak conductive plane M_2_, which differ in conductivity. The M_1_ is coated around M_2_, and has good contact with M_2_. The conductivity of M_1_ is much higher than the conductivity of M_2_. Two electrodes were arranged on the M_1_ and a DC bias voltage was added onto the two electrodes. If a uniform electric field can be generated in M_2_, the potential distribution in M_2_ will be linear with the position in the direction of the electronic field. The coordinate of any point in M_2_ can be obtained depending on the linear relationship between the potential and position of this point if the potential of this point can be detected by some means. Therefore, the key problem of this tactile sensor is how to realize the uniform electric field in a conductive plane with any irregular shape. The middle layer L2 is an isolated layer composed of an evenly distributed insulated isolated point array.

**Figure 2:**
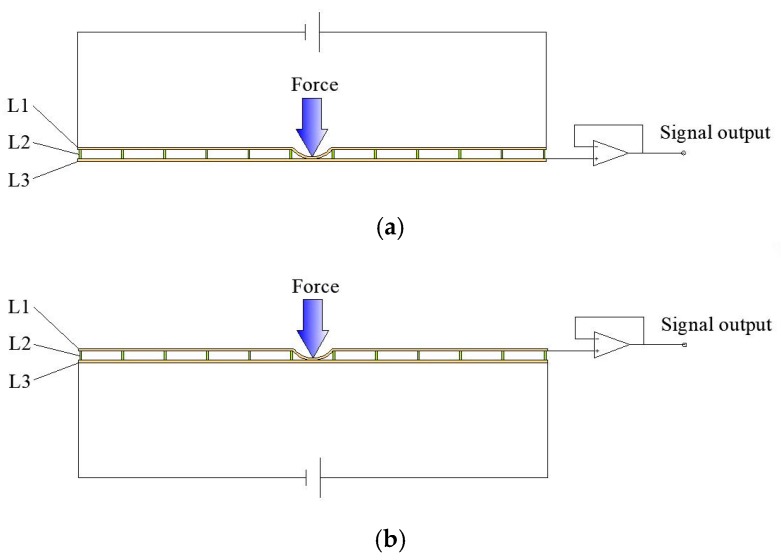
Cross-section of the sensor. (**a**) Position detection of the X axis. (**b**) Position detection of the Y axis.

The potential of any point in M_2_ can be detected as followed. A DC bias voltage is first applied between the two electrodes of the upper Layer L1, and a uniform electric field will be generated in M_2_ of layer L1. If an external force is applied to the surface of the sensor, the upper layer L1 will be deformed, penetrating through the middle layer L2, and making contact with the lower layer L3 at the contact point, as shown in Figure 2. Then, the potential of the lower layer L3 is equal to that at the contact point of the upper layer L1, on the condition that the lower layer L3 has access to an operational amplifier with infinite input impedance in theory. Similarly, if a DC bias voltage is applied between the two electrodes of the lower layer L3, it is also easy to obtain the potential of the contact point by means that the upper layer L1 is accessed by the operational amplifier. The uniform electric field in the two conductive layers L1 and L3 can be generated alternately and perpendicular in direction. This makes it possible to obtain the X and Y coordinates of any point in M_2_.

Next, the key is to construct a uniform electric field in a flexible conductive plane of any shape.

### 2.2. Construction of the Uniform Electric Field

First, two electrodes were assumed to be arranged at both sides of an irregularly shaped homogeneous conductive plane consisting of only one material. If a DC bias voltage is applied between two electrodes, a constant current field will be formed in the conductive plane. Since the interior of the homogeneous conductive plane is a passive region, the potential distribution in the conductive plane should satisfy the Laplace equation:

(1) $$\nabla^{2}\varphi =0.$$

**Figure 3:**
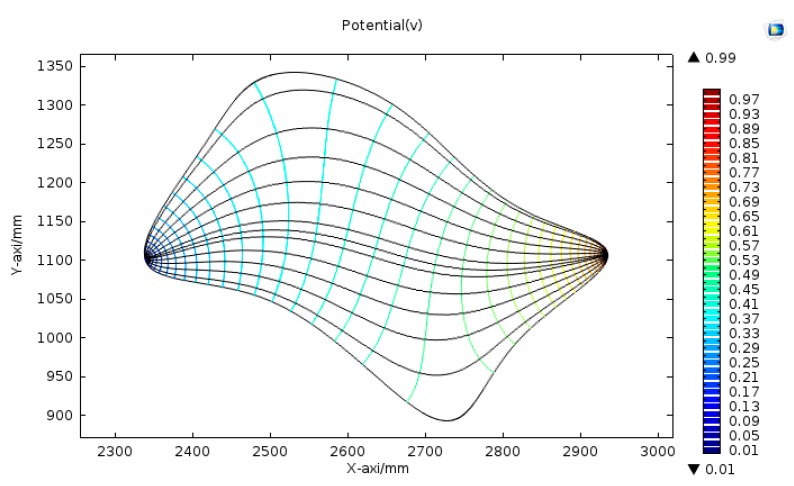
Electric field distribution and potential distribution in the conductive plane.

Setting the voltage between the two electrodes to 1 V, the electric field distribution and potential distribution in the conductive plane are shown in Figure 3.

As shown in Figure 3, the electric field generated is not uniform. However, the electric field generated in the conductive plane is constant, which has the same properties as the electrostatic field. According to the uniqueness theorem of the electrostatic field, if the potential distribution of the boundary of the conductive plane is determined, the electric field in the conductive plane will be uniquely determined. By reasonably constructing the potential distribution of the boundary of the conductive plane, the uniform electric field can be generated in the conductive plane.

**Figure 4:**
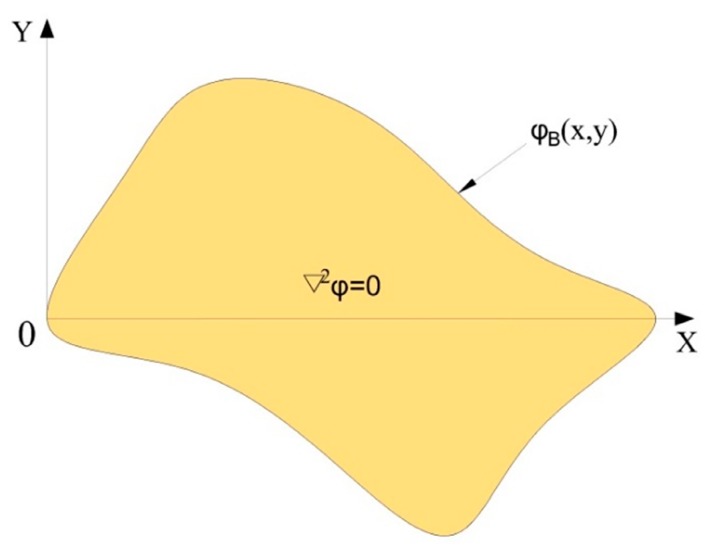
Irregular shaped homogeneous conducting plane in the rectangular coordinate system.

As shown in Figure 4, a rectangular coordinate system was established in the homogeneous conductive plane with an irregular shape.

**Figure 5:**
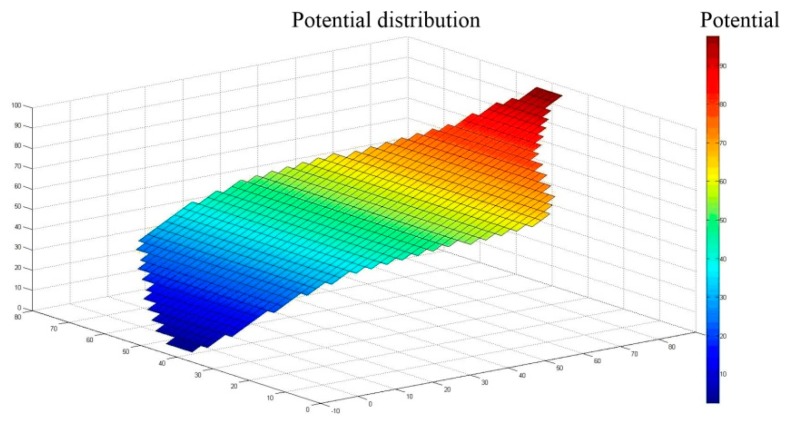
Potential distribution of the conductive plane.

Setting the boundary potential distribution to $\varphi_{B}(x,y)=cx$, where c is a positive constant, represents the magnitude of the change in the potential gradient in the uniform electric field. The larger the c, the more obvious the potential change. MATLAB was used to solve the Laplace equation under the boundary conditions (we set the boundary potential distribution to $\varphi_{B}(x,y)=x$), and the potential distribution in the conductive plane is shown in Figure 5.

From Figure 5, it can be seen that a gradient linear potential distribution formed in the irregularly shaped conductive plane. Therefore, for any homogeneous conductive plane in a rectangular coordinate system, if the boundary potential is $\varphi_{B}(x,y)=cx$, the conductive plane will generate a uniform electric field along the X axis, and form a gradient linear potential distribution along the X axis. In the same way, if the boundary potential is $\varphi_{B}(x,y)=cy$, the conductive plane will generate a uniform electric field along the Y axis and form a gradient linear potential distribution along the Y axis.

To this end, it is important to know how to construct the boundary potential $\varphi_{B}(x,y)=cx$ and $\varphi_{B}(x,y)=cy$ at an irregular plane shape.

### 2.3. Construction of the Boundary Potential Distribution Meeting $\varphi_{B}(x,y)=cx$

In this paper, two conductive materials with different conductivities were used to construct the conductive plane, as shown in Figure 1. The two conductive materials should have good flexibility, and the conductivity of one is much greater than that of the other one. A flexible weak conductive layer was made of the material with low conductivity, and a special designed strong conductive line made of the material with high conductivity was arranged along the edge of the weak conductive layer. Thus, the conductive plane is constructed.

**Figure 6:**
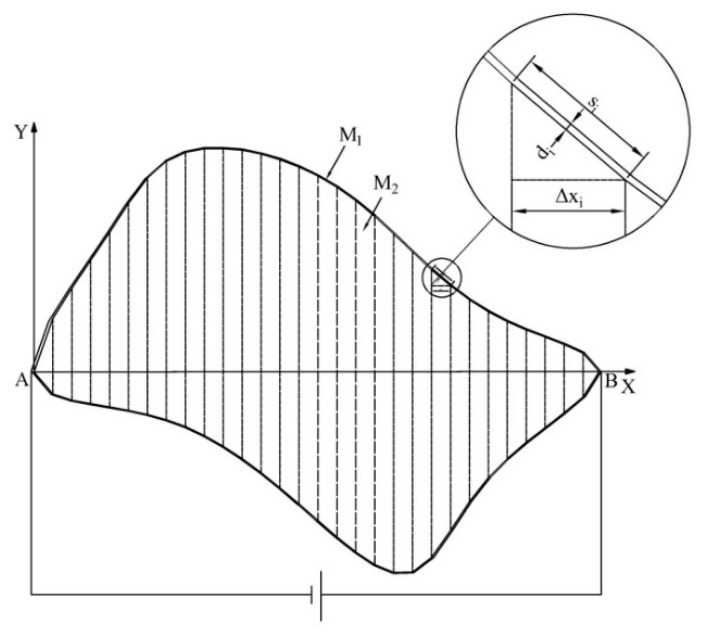
The structure of the irregular conducting plane.

The irregular plane shown in Figure 3 was used as an example to construct the boundary potential. First, the boundary curve of the irregular plane was discretized to a polygon model. Then, the polygon model was built in a rectangular coordinate system. Finally, the two conductive materials, M_1_ and M_2_, were used to construct the polygon conductive plane, as shown in Figure 6.

The conductivity of M_1_ was much higher than that of M_2_, and M_1_ had good contact with M_2_. The width $d_{i}$ of each segment of the ring-shaped conductive layer M_1_ satisfies

(2) $$d_{i}=\frac{s_{i}}{\Delta x_{i}}d_{0},$$

where $s_{i}$ is the side length of the polygon corresponding to the $\Delta x_{i}$ segment, $d_{0}$ is the reference width and $d_{0}\ll \mathrm{AB}$. Assuming that the distribution of the conductivity inside M_1_ is uniform, the resistance $R_{i}$ of each segment of M_1_ satisfies

(3) $$\frac{R_{i}}{R_{total}}=\frac{\Delta x_{i}}{2\mathrm{AB}},$$

where $R_{total}$ is the sum of the resistances of the segments in M_1_. If a constant bias voltage *U* is applied between A and B, since the conductivity of M_1_ is much greater than that of M_2_, most of the current will flow from the positive electrode B through the ring-shaped M_1_ to the negative electrode A. According to the distribution of the resistance between A and B, the potential distribution of A to B in M_1_ is

(4) $$\phi_{\mathrm{AB}}(x,y)=\frac{U}{\mathrm{AB}}x.$$

Equation (4) satisfies the boundary potential requirement of the uniform electric field proposed in this paper. Since M_1_ was in good contact with M_2_, the boundary potential distribution of M_2_ also satisfied Equation (4). As a result, a boundary potential distribution that generates a uniform electric field was built in M_2_ of the polygon conductive plane.

### 2.4. Contact Point Position Detection

**Figure 7:**
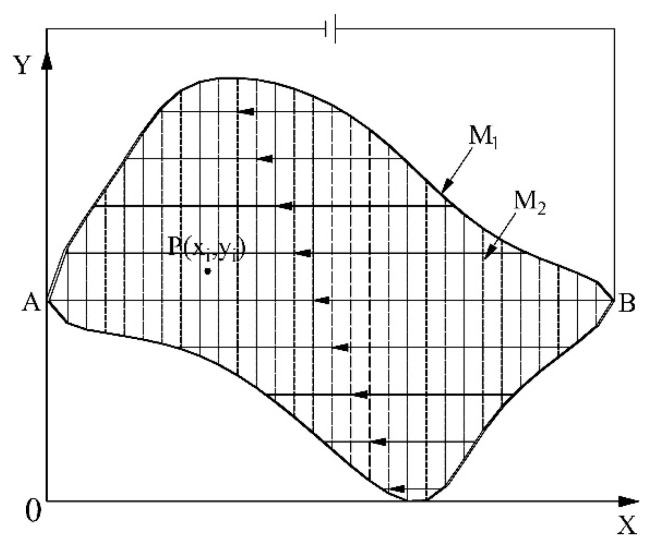
X-axis detecting principle.

When a constant bias voltage is applied across the electrodes of the conductive plane A, a uniform electric field along the X axis is generated, and a gradient linear potential distribution along the X axis is formed in M_2_, as shown in Figure 7.

In Figure 7, the solid lines with arrows are electric field lines and dotted lines are equipotential lines. The potential distribution of the planar area can be expressed as

(5) $$\varphi_{1(x,y)}=f_{1}(x,y).$$

**Figure 8:**
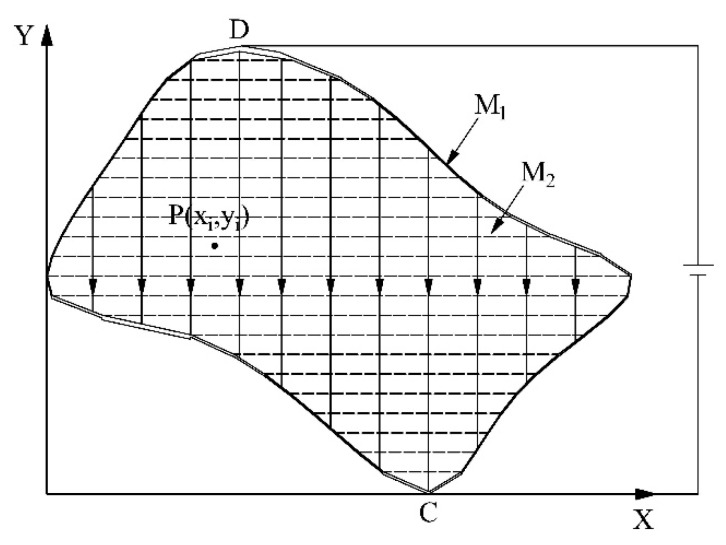
Y-axis detecting principle.

Similarly, when a constant bias voltage is applied across the electrodes of the conductive plane B, a uniform electric field along the Y axis is generated, and a gradient linear potential distribution along the Y axis is formed in M_2_, as shown in Figure 8.

In Figure 8, the solid lines with arrows are electric field lines and dotted lines are equipotential lines. The potential distribution of the planar area can be expressed as

(6) $$\varphi_{2(x,y)}=f_{2}(x,y).$$

By measuring the potential of point $p(x_{i},y_{i})$ in two electric fields $\varphi_{1}$ and $\varphi_{2}$, the position coordinates of the point can be calculated. Its potential can be expressed as

(7) $$\left[\begin{array}{c} \varphi_{1}\\ \varphi_{2} \end{array}\right]=\left[\begin{array}{c} f_{1}(x_{i},y_{i})\\ f_{2}(x_{i},y_{i}) \end{array}\right].$$

Since the value of the potential in the direction of the electric field is proportional to its distance from the electrode, the positional coordinates of the contact point can be easily calculated by measuring the potential $\varphi_{1}$, $\varphi_{2}$ of the contact point in the two electric fields. Its position coordinates can be expressed as

(8) $$\left[\begin{array}{c} x\\ y \end{array}\right]=\left[\begin{array}{c} g_{1}(\varphi_{1})\\ g_{2}(\varphi_{2}) \end{array}\right],$$

where $g_{1}(\varphi_{1})$ and $g_{2}(\varphi_{2})$ are the inverse function of $f_{1}(x_{i},y_{i})$ and $f_{2}(x_{i},y_{i})$, respectively.

When the conductive plane is bent, the density of the conductive medium caused by bending is minimal because the thickness of the conductive medium is very small. It is considered that the electric field distribution in the conductive plane under the bending condition is invariant.

## 3. Finite Element Analysis of Conductive Plane

### 3.1. Influence of the Ratio of the Conductivity of M_1_ to M_2_

**Figure 9:**
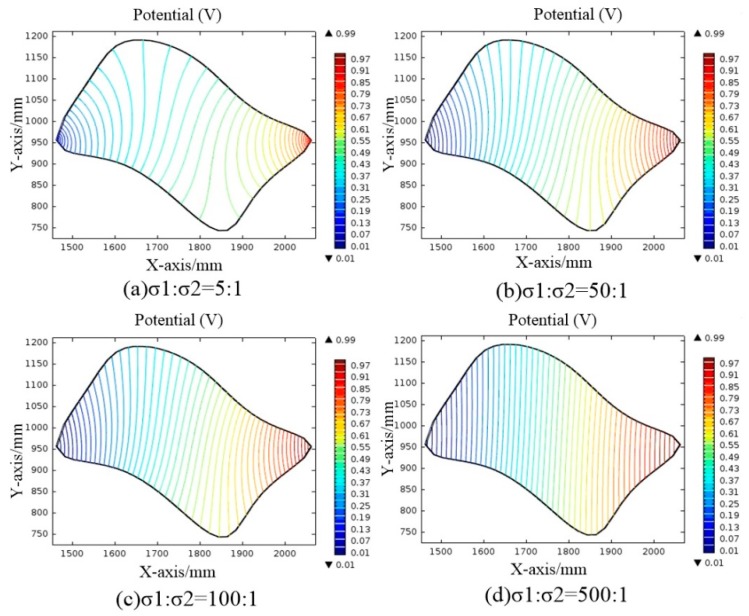
Potential distribution in the conductive plane with different conductivity ratios.

Under the same conditions, the ratio of the conductivity of M_1_ to M_2_ will affect the potential distribution in the conductive plane. COMSOL Multiphysics was used to simulate the conductive plane with different ratio of the conductivity of M_1_ to M_2_. The potential distribution of the conductive plane is shown in Figure 9.

As shown in Figure 9, the greater the ratio of $\sigma_{1}/\sigma_{2}$, the more uniform the electric field distribution in the conductive plane. If the ratio of the conductivity of M_1_ to M_2_ is $\sigma_{1}/\sigma_{2}\geq 500$, a relatively uniform electric field distribution can be formed in M_2_.

### 3.2. Simulation of Different Shaped Tactile Sensors

**Figure 10:**
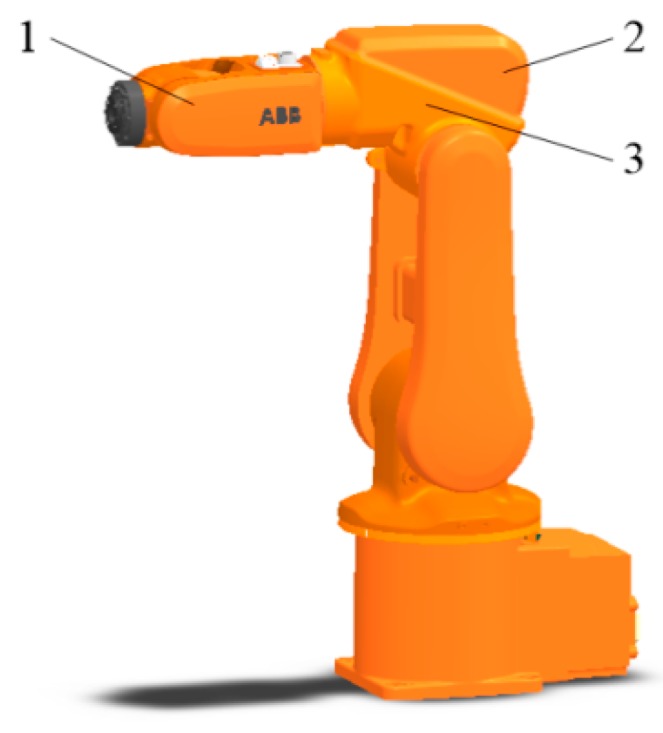
Model of the IRB120 robot.

**Figure 11:**
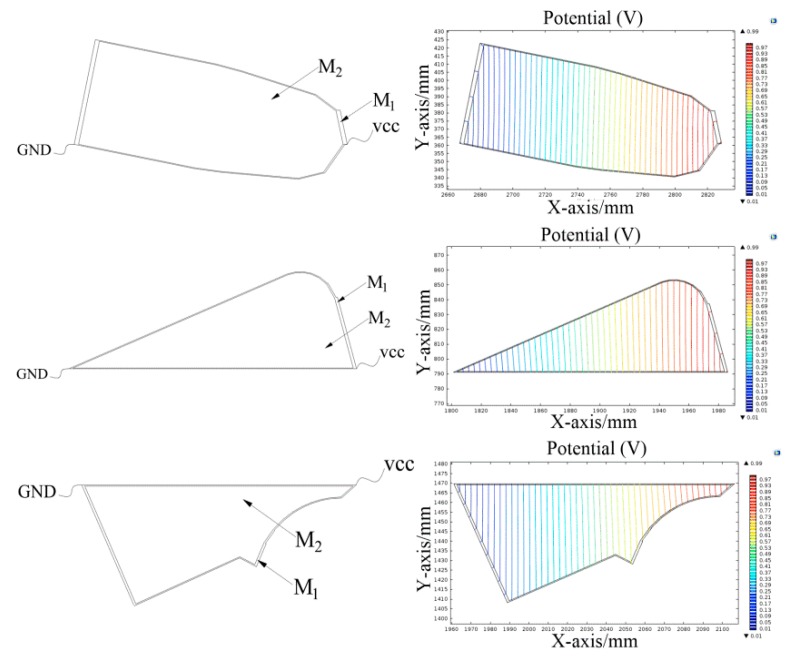
The simulation of constant current field of the conductive plane.

As shown in Figure 10, three kinds of irregular shapes were randomly selected from the surface of the IRB120 robot. The conductive planes of the tactile sensor were constructed using the method proposed in this paper. The simulation of the constant current field was performed using COMSOL Multiphysics. We set the ratio of conductivity of M_1_ to M_2_ at 500:1, and 1 V voltage was applied between the electrodes at the two ends. The structural model and potential distribution of the conductive plane are shown in Figure 11.

As shown in Figure 11, a linear gradient potential distribution formed in M_2_ when the electrodes at different ends of the conductive plane were applied with a constant bias voltage.

## 4. Experiments

### 4.1. Sample Production

**Figure 12:**
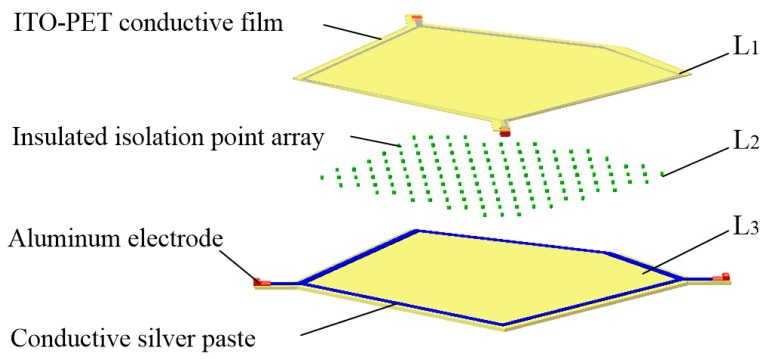
Structural model of tactile sensor.

A flexible irregular pentagon sensor sample was fabricated based on the construction method of the conductive plane proposed in this paper. The structural model is shown in Figure 12.

**Figure 13:**
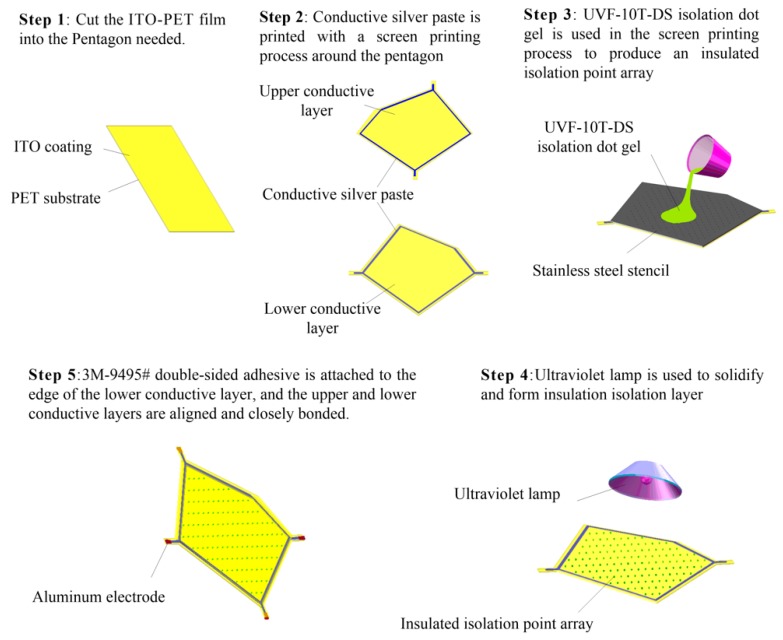
Process of fabricating the sensor.

**Figure 14:**
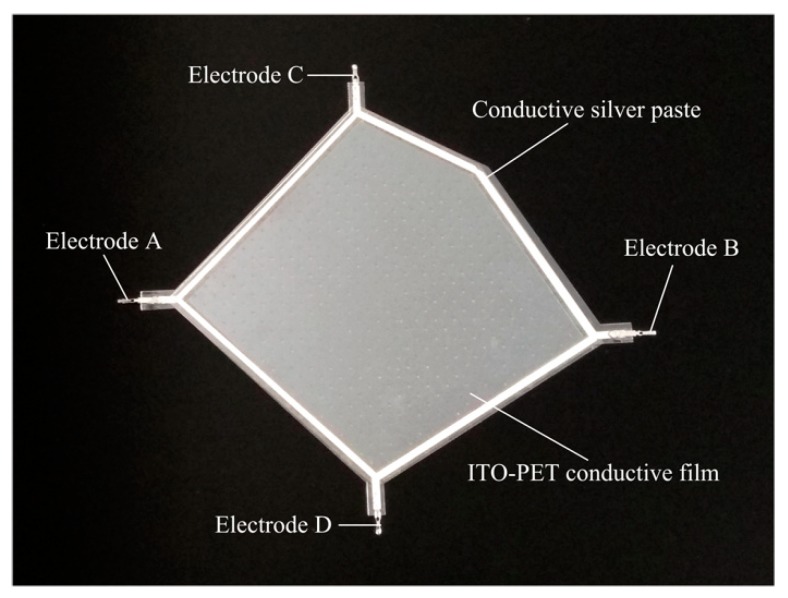
Sensor sample.

**Figure 15:**
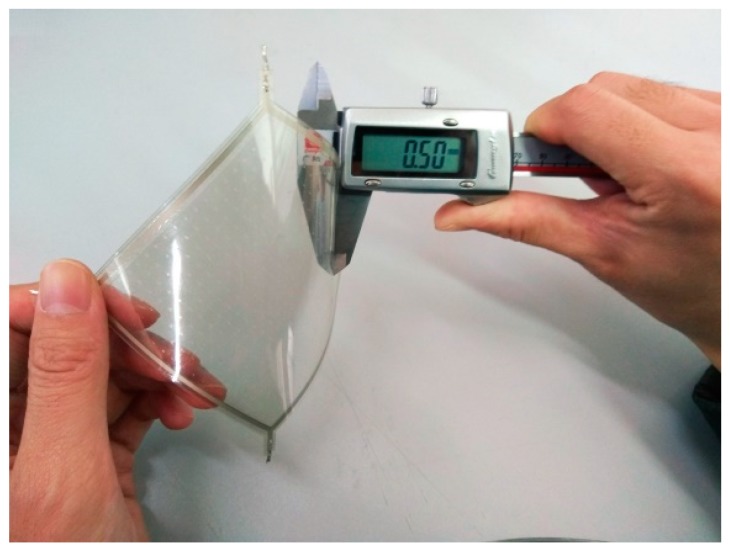
Thickness of the sensor sample.

The upper layer L1 and the lower layer L3 were pentagonal conductive layers consisting of an ITO-PET conductive film with lower conductivity and a pentagonal flexible circuit printed with a conductive silver paste with higher conductivity at the edges. Metal electrodes were arranged at both ends of the flexible circuit. The middle layer L2 was an isolating layer consisting of an evenly distributed insulated isolated point array. As shown in Figure 13, the sensor was made as follows:

(1) Upper layer L1: An ITO-PET conductive film with sheet resistance of 100 Ω/cm^2^ and a thickness of 0.175 mm (where the thickness of the ITO film was 70 nm) was cut into a pentagon. A conductive silver paste with a conductivity of about 2.4 × 10^7^ S/m was printed using a screen printing process around the pentagon sides onto ITO-PET conductive film. A pair of aluminum electrodes were fitted at both corners of the pentagon.
(2) Isolating layer L2: UVF-10T-DS isolation dot gel was used in the screen printing process to produce an insulated isolation point array with a diameter of 0.3 mm, a spacing of 4 × 4 mm, and a height 0.1 mm in the upper composite conductive layer. An ultraviolet lamp was used to solidify and form the insulation isolation layer.
(3) Lower layer L3: This was similar to the upper layer L1 and was also composed of the ITO-PET conductive film and the conductive silver paste. However, the shape of the strong conductive line printed by the conductive silver paste was different to that of the upper layer L1.
(4) Assembling: 3M-9495# double-sided adhesive was attached to the edge of the lower conductive layer, and the upper and lower conductive layers were aligned and closely bonded. The sensor sample is shown in Figure 14 and Figure 15.

### 4.2. Contact Experiment of Tactile Sensor

**Figure 16:**
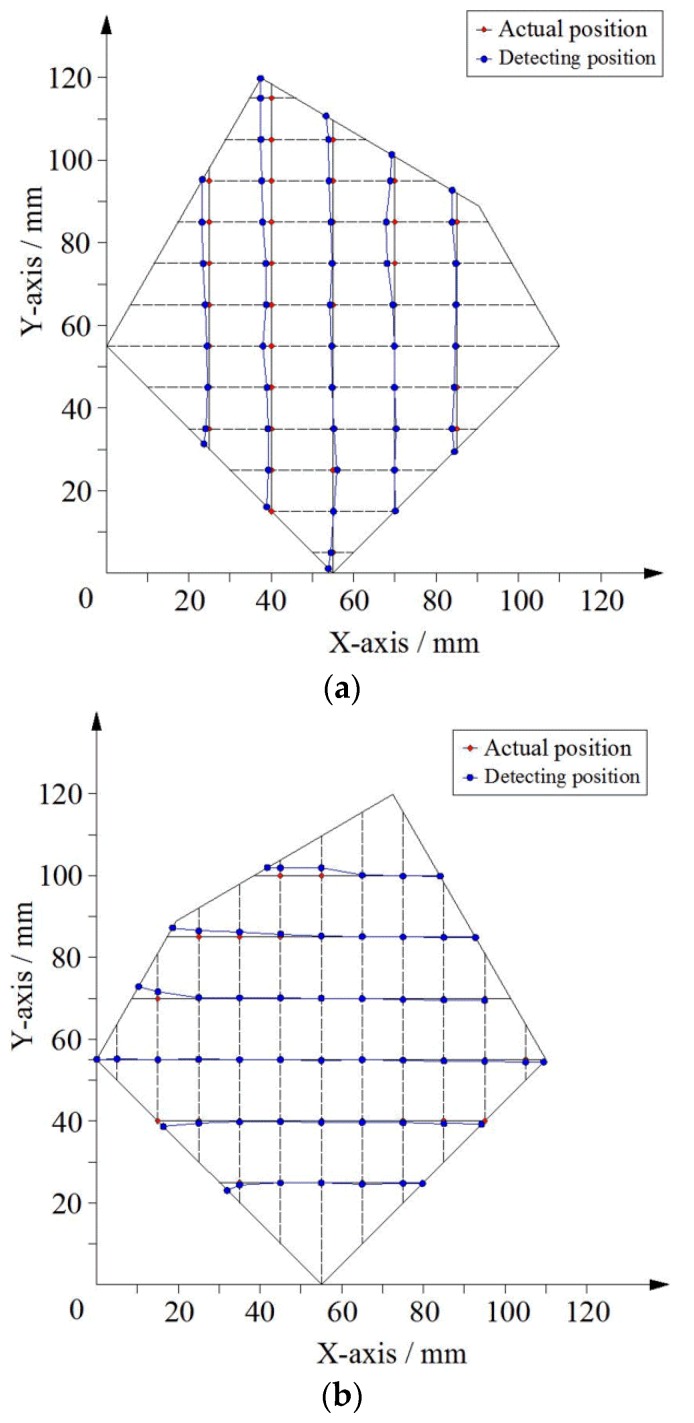
Experimental results for point contact. (**a**) Bias voltage applied in the direction of X-axis. (**b**) Bias voltage applied in the direction of Y-axis.

The sensor’s conductive layer was used for point contact position detection. Using the GPD-3303S programmable linear DC power supply provided a DC bias voltage, and the voltage between the electrodes was set to 500 mV. When the bias voltage was applied between the two electrodes in the upper conductive layer, a series of points on the line *x* = 25 mm, *x* = 40 mm, *x* = 55 mm, *x* = 70 mm, *x* = 85 mm were selected and pressed directly by the voltmeter probe (as point contact) and the value of the voltage was readout. Similarly, when the bias voltage was applied between the two electrodes in the lower conductive layer, a series of points on the line *y* = 25 mm, *y* = 40 mm, *y* = 55 mm, *y* = 70 mm, *y* = 85 mm, *y* = 100 mm were selected and pressed directly by the voltmeter probe (as point contact) and the value of the voltage was readout. The value of the measured voltage was converted into a position coordinate using Equation (8), and the position coordinates of the calculated position were compared with the actual position coordinates. The experimental results are shown in Figure 16.

As shown in Figure 16, the maximum position error in the X-axis direction within the detection area was not more than 3.6 mm, and the maximum position error in the Y-axis direction within the detection area was not more than 2.1 mm. The main cause of the error was because the silver paste circuit had a certain degree of unevenness, and the voltage drop distribution of the circuit was not uniform, resulting in the non-uniform gradient distribution of the potential in the conductive plane. In addition, the uneven distribution of the sheet resistance of the ITO film also led to the uneven distribution of the potential in the local area of the conductive plane.

**Figure 17:**
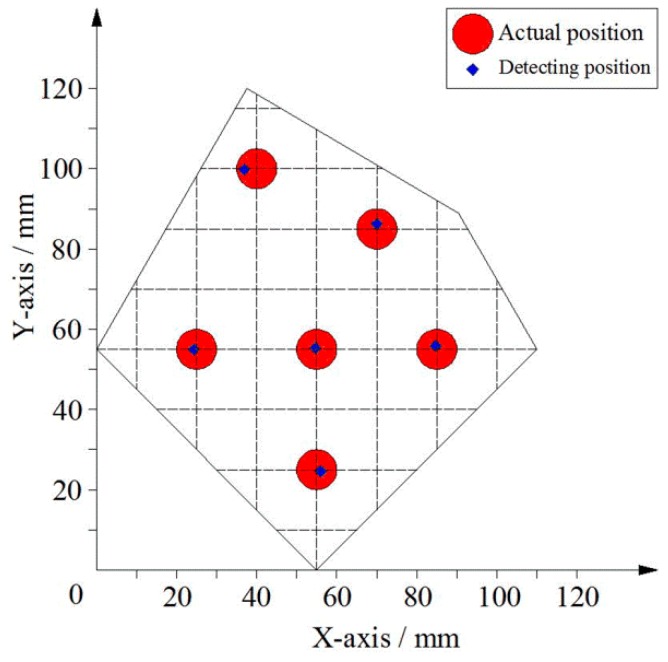
Experimental results for the area contact.

When the contact area was not a point, but an area, such as a circular area with a radius of 5 mm, six circular areas on the plane were selected as the measuring points. The comparison of the position coordinates of these points with the actual position (the coordinates of the center of the center) is shown in Figure 17.

As shown in Figure 17, the error of the area contact position of the six circular regions was less than the radius of the contact area, and the measured position was still in the contact area.

### 4.3. Signal Processing of Tactile Sensor

**Figure 18:**
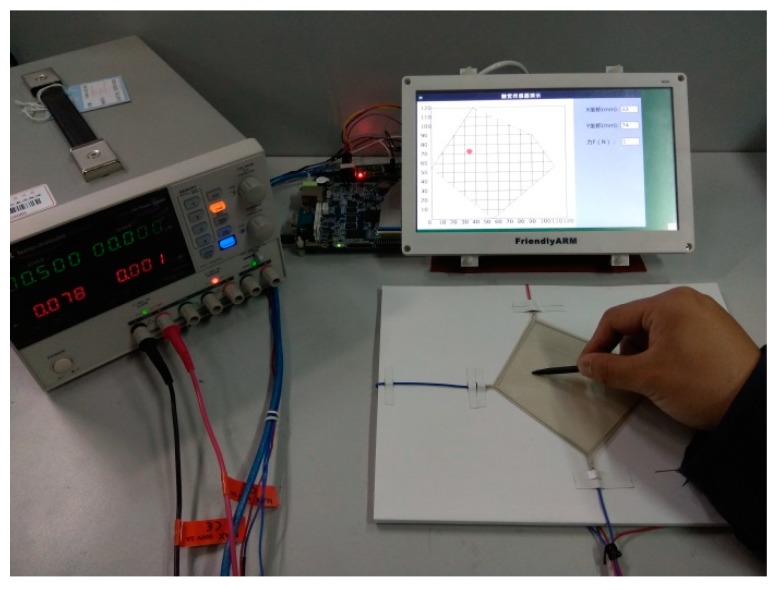
Position detection on the plane.

**Figure 19:**
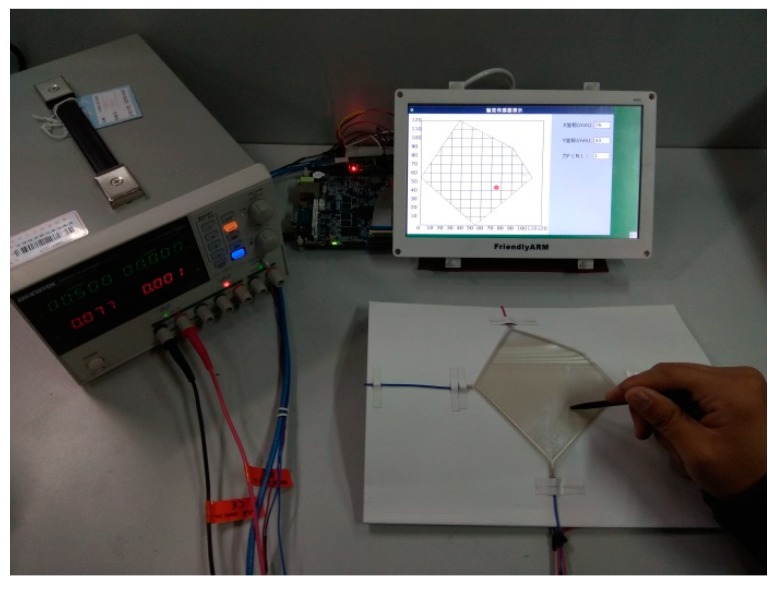
Position detection on the curved surface.

Based on the PIC18F25K80 chip, we built a signal processing circuit of the tactile sensor, which could carry out the switching of the dual DC voltage steady voltage input to the sensor, and the functions of acquisition, amplification, filtering, A/D conversion, and external communication of the output signal. Using the Mini210s hardware system based on the CortexTM-A8 processor and the Qt Creator integrated development environment, the output display module of the tactile sensor was developed, and the dynamic display function of the sensor’s contact position information was realized. The tactile sensor was attached to the plane paperboard and the curved paperboard, respectively, for contact position detection. The experimental devices are shown in Figure 18 and Figure 19.

The experiment showed that the sensor system could display the contact position information on the display screen in real time when a touch pen with a diameter of 2 mm was used for contact on the sensor attached to the curved surface with a radius of 300 mm.

The minimum contact force needed for the sensor to be able to detect the contact position was approximately 0.9 N, which is related to the stiffness of the ITO-PET film and the spacing of the insulated isolated point array. The minimum contact force could be further reduced by using an ITO-PET film with a smaller thickness and appropriately increasing the spacing of the insulated isolated point array.

In addition, the bending performance of the sensor was further tested. The experiment showed that when the radius of curvature of the sensor reached 20 mm, the insulated isolated point array could effectively isolate the upper and lower conductive layers, and the sensor system could still work normally.

The tactile sensor proposed in this paper is suitable for single point contact detection. When two points, or more, are pressed (such as using fingers) at the same time at different positions of the sensor, the sensor can only sample one set of potential signals. Such signals cannot represent any pressed point. Therefore, the sensor can only detect the position of single point contact.

Finally, it is worth pointing out that the tactile sensor can be easily fabricated for a large scale. Theoretically, the size of the tactile sensor can be unlimited. For large scale, it should be pointed out that with the increase of sensor size, the difficulty of screen printing method will also increase, and the sensor resolution will also be decreased to some extent.

## 5. Conclusions

Based on the uniqueness theorem of the electrostatic field, a novel flexible film tactile sensor that can detect contact position and be made into any plane shape was proposed in this paper. Based on the structural design of the conducting plane, we realized the construction of a uniform electric field in the arbitrary shape conductive plane. We established the theoretical model of the sensor and analyzed the influence on the electric field distribution for conductive layers of different shape. A sensor for three layers of thin film structure was proposed. The sensor samples were made. The point contact and area contact experiments of the sensor samples were carried out. A tactile sensor signal processing circuit was built, and the dynamic display experiment of contact position detection was carried out. The experimental results showed that the principle of the sensor is feasible. The sensor had a simple structure, light, good flexibility, and easily covered the surface of the robot in large areas, and the signal processing was simple. Therefore, it can provide necessary information support for safe human–robot interactions.
